# Changes in the Profile of Antibiotic Prescriptions by Dentists in Brazil during the Pandemic

**DOI:** 10.1155/2022/6570812

**Published:** 2022-06-06

**Authors:** Fernando de Sá Del Fiol, Isaltino Pereira de Andrade-Jr, Marcus Tolentino da Silva, Silvio Barberato-Filho, Luciane Cruz Lopes, Cristiane de Cassia Bergamaschi

**Affiliations:** Doctoral Program in Pharmaceutical Sciences, University of Sorocaba, Sorocaba, Brazil

## Abstract

During the COVID-19 pandemic, people worldwide, including the scientific community, were insecure and fearful. The lack of vaccines at the beginning of the pandemic and the high mortality rate led to a search for alternative treatments for COVID-19. Among these proposals, a postulated activity of azithromycin was frequently studied in early treatment. In view of this, many countries saw an increase in the consumption of this antibiotic. Thus, the objective of this study was to evaluate, in Brazil, whether there was an increase in azithromycin prescriptions made by dentists, as they may have been prescribing this antibiotic as a probable treatment for COVID-19. This is an interrupted time series that analyzed antimicrobial prescriptions data between January 2014 and July 2021. The data were taken from the National System of Controlled Products Management, and pre- and postpandemic periods were compared. To assess changes in azithromycin consumption, Joinpoint regression and analysis of variance, followed by Dunnett's test, were used. More than 38 million prescriptions written during the period were analyzed. Amoxicillin (72.3%), azithromycin (18.0%), cephalexin (6.1%), and metronidazole (3.58%) were the most prescribed antibiotics. At the beginning of the pandemic, there was a drop in amoxicillin prescriptions motivated by a decrease in consultations, but conversely, in less than three months, azithromycin prescriptions grew by more than 100%. The exaggerated use of this antibiotic during the pandemic will certainly have consequences in the short and medium term on indicators of bacterial resistance. The use of guidelines and respect for the therapeutic protocols of government agencies should be fundamental for collective and strategic action in the fight against health emergencies.

## 1. Introduction

The COVID-19 pandemic has produced countless feelings of insecurity and fear in the world population. In early 2020, the lack of a vaccine and uncertainties about the evolution of the disease led the scientific community to search for early treatments for COVID-19, seeking to reposition drugs [1–4]. One of these drugs with potential action against the coronavirus was azithromycin. Its immunomodulatory and anti-inflammatory effects [5] were responsible for positioning the antibiotic as a possible adjuvant in the treatment of the disease. With this perspective, some studies have shown that, despite the lack of proof of the effectiveness of the antibiotic in the treatment of COVID-19, there was an increase in the demand and consumption of this antibiotic [6–9]. In Brazil, since 2013, all antibiotic prescriptions have been registered in a national drug sales control system known as SNGPC. This system provides information about the antibiotic, the professional who prescribed it (doctor, dentist, or veterinarian), and the patient [10]. The search for treatments and the insecurity of the population pressured health professionals to prescribe treatments without scientifically proven effectiveness [11–13], endangering the health of the population, and in the case of antibiotics, increasing the exposure of azithromycin to microorganisms, leading to an increase in their levels of resistance [14, 15]. With these data, the present study sought information on behavior and trends in antibiotic prescriptions made by dentists in Brazil before and during the pandemic.

## 2. Methods

This study was an interrupted time series that analyzed trends of antibiotic prescriptions made by dentists in Brazil before and during the pandemic period (2014–2021). Data were collected in the National System of Controlled Products Management (SNGPC). This system shows the number of antibiotics sold, the professional registration number of the prescriber, and the data of the patient prescribed the antibiotic [10].

### 2.1. Statistical Methods

Data from the four oral antibiotics most commonly prescribed by dentists in Brazil were analyzed. The data were calculated as monthly averages, year by year, and analysis of variance was applied, followed by the Dunnett Multiple Comparisons Test (GraphPad Instat—version 3.05), which used the pandemic period as a control for comparison with other years (2014–2019). To assess the changes in monthly trends in the use of azithromycin, we applied Joinpoint regression, a statistical method used to identify the best-fitting points in the case of the presence of a statistically significant change in the trend [16]. The Joinpoint Regression Program was used (version 4.9.0.0, March 2021; Statistical Research and Applications Branch, National Cancer Institute).

## 3. Results

**Figure 1:**
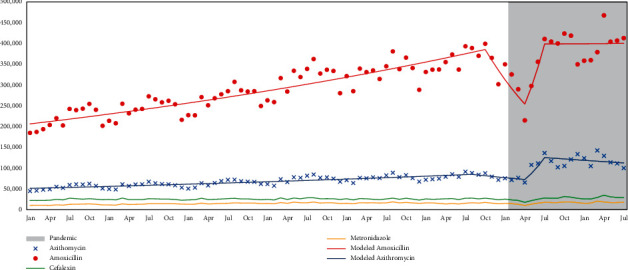
Evolution of azithromycin, amoxicillin, cephalexin, and metronidazole prescriptions between 2014 and 2021. Modeled regression analysis (Joinpoint regression) for amoxicillin and azithromycin and pandemic period (in gray).

A total of 38,469,592 prescriptions were analyzed in the period studied (2014–2021). The antibiotics most prescribed in Brazil by dentists between 2014 and 2021 were amoxicillin (with or without clavulanic acid) (72.3%), azithromycin (18.0%), cephalexin (6.1%), and metronidazole (3.58%). The data in Figure 1 show the evolution, year by year, in the prescribing of these antibiotics by dentists in Brazil. The figure also shows (in gray) the period of the beginning of the pandemic in Brazil.

In the first months of the pandemic and lockdown, the Joinpoint regression analysis (Figure 1, red lines) shows a monthly percent change (MPC), revealing a significant drop in amoxicillin prescriptions of around 6.71% per month. This drop can be attributed to the fear of contamination and uncertainties that dominated the beginning of the pandemic. Other studies have shown that visits to medical offices significantly decreased during that period, with a consequent decrease in the prescription of antimicrobials [17–20]. Conversely, during the months of April to July 2020, with the lack of vaccines and the supposed therapeutic activity of azithromycin against COVID-19, our data show a statistically significant MPC: a 19.92% monthly increase between April and July 2020 (blue lines). Sales of approximately 65,000 units in April reached 136,000 units in July, an increase of over 100% in just three months. Nothing in Brazil's oral health situation could justify this increase. The profile of azithromycin prescriptions did not show any significant variation from January 2014 to April 2020. Therefore, the search for a treatment for COVID-19 through the use of azithromycin prescribed by dentists must be the explanation for this abrupt and unique increase. Cephalexin and metronidazole prescriptions did not change before or during the pandemic, making them stable throughout the study period.

Table 1 shows the monthly averages of antibiotic sales, year by year and during the pandemic. Amoxicillin, cephalexin, and metronidazole did not show any (statistically significant) increase during the pandemic months when compared to 2018 and 2019. Azithromycin consumption, on the other hand, showed an increase during the pandemic (*p* < 0.001) when compared to all years studied (2014–2019). Another fact that calls attention is the participation (%) of each antibiotic during the years studied. Azithromycin historically represented 16 to 17% of antibiotic sales between 2014 and 2019. During the pandemic, it represented 20.69% of sales of antibiotics prescribed by dentists in Brazil. Conversely, amoxicillin, which accounted for 73.7% and 74.5% of monthly sales in 2018 and 2019, respectively, decreased its share to 70.9% during the pandemic.

A study conducted in Australia also evaluated changes in dental prescriptions. The results showed a decrease in antibiotic prescribing in the same period found in the present study, with the lowest prescribing indicators in April 2020. Data from the Australian study do not cite azithromycin as a medication used by dentists; instead, amoxicillin (77%), metronidazole (13%), clindamycin (5%), and cephalexin (3%) are cited. Other antibiotics accounted for 2% of Australian prescriptions [21].

It is important to emphasize that changes in antibiotic prescription patterns can significantly alter the oral and surrounding tissue's microbiota, creating more favorable conditions for the emergence or resurgence of oral infections, such as periodontal infections [22].

## 4. Conclusions

The decrease found in amoxicillin prescriptions at the beginning of the pandemic is a strong indicator of decreases in dental appointments. This occurred at the beginning of the pandemic (April 2020), when uncertainties about contamination and care were still very high. Vaccinations and advances in medical care brought a return to dental appointments with the return of prescriptions. In the case of azithromycin, it seems very clear that its use took place as a supposed “treatment for COVID-19” prescribed by dentists. It is essential that all prescribing health professionals (doctors, dentists, and veterinarians) base their prescriptions on official guidelines and safety guidelines from official health bodies. The indiscriminate use of azithromycin has not shown any effectiveness in fighting the disease and will certainly have an impact on the indicators of antimicrobial resistance.

## Figures and Tables

**Table 1:** Monthly commercial units sold (mean), standard deviation (SD), sales share (%), and *p* value of amoxicillin, azithromycin, cephalexin, and metronidazole before and during the pandemic period in Brazil, per year.

Year	Azithromycin				Amoxicillin				Cephalexin				Metronidazole				Total		
	Mean	SD	*p* value	Sales share (%)	Mean	SD	*p* value	Sales share (%)	Mean	SD	*p* value	Sales share (%)	Mean	SD	*p* value	Sales share (%)	Mean	SD	*p* value
2014	54.003	6.28	<0.001	17.52	217.817	24.994	<0.001	70.67	24.453	1.92	n.s.	7.93	11.957	1.298	<0.001	3.88	308.230	34.213	<0.001
2015	58.698	5.623	<0.001	17.25	243.291	21.682	<0.001	71.48	24.999	1.404	n.s.	7.35	13.366	1.174	<0.001	3.93	340.353	29.148	<0.001
2016	64.069	6.912	<0.001	17.18	268.374	24.946	<0.001	71.96	25.480	1.334	n.s.	6.83	15.033	1.134	n.s.	4.03	372.955	33.779	<0.001
2017	73.118	8.204	<0.001	17.05	312.919	33.179	<0.001	72.97	26.435	1.762	n.s.	6.16	16.387	1.329	n.s.	3.82	428.859	43.962	<0.001
2018	76.482	6.76	<0.001	16.98	332.060	27.612	n.s.	73.70	25.621	1.451	n.s.	5.69	16.393	1.185	n.s.	3.64	450.556	36.492	<0.001
2019	80.561	6.774	<0.001	16.80	357.432	29.393	n.s.	74.53	26.108	1.245	n.s.	5.44	15.493	1.158	n.s.	3.23	479.593	37.564	n.s.
Pandemic	107.859	22.32		20.69	369.855	58.585		70.96	27.083	3.981		5.20	16.415	2.404		3.15	521.212	80.677	

## Data Availability

The data that support the findings of this study are openly available in Brazilian Data Portal at https://dados.gov.br/dataset (https://dados.gov.br/dataset/venda-de-medicamentos-controlados-e-antimicrobianos-medicamentos-industrializados).
